# Training of front-line health workers for tuberculosis control: Lessons from Nigeria and Kyrgyzstan

**DOI:** 10.1186/1478-4491-6-20

**Published:** 2008-09-29

**Authors:** Niyi Awofeso, Irina Schelokova, Abubakar Dalhatu

**Affiliations:** 1 School of Public Health and Community Medicine, University of New South Wales, Sydney 2052, Australia; 2 School of Population Health, University of Western Australia, Perth, Australia; 3 National Tuberculosis Institute, Bishkek, Kyrgyzstan; 4 Field Training Unit, National Tuberculosis and Leprosy Training Centre, Zaria, Nigeria

## Abstract

Efficient human resources development is vital for facilitating tuberculosis control in developing countries, and appropriate training of front-line staff is an important component of this process. Africa and Central Asia are over-represented in global tuberculosis statistics. Although the African region contributes only about 11% of the world population, it accounts for at least 25% of annual TB notifications, a proportion that continues to increase due to poor case management and the adverse impact of HIV/AIDS. Central Asia's estimated current average tuberculosis prevalence rate of 240/100 000 is significantly higher than the global average of 217/100 000. With increased resources currently becoming available for countries in Africa and Central Asia to improve tuberculosis control, it is important to highlight context-specific training benchmarks, and propose how human resources deficiencies may be addressed, in part, through efficient (re)training of frontline tuberculosis workers. This article compares the quality, quantity and distribution of tuberculosis physicians, laboratory staff, community health workers and nurses in Nigeria and Kyrgyzstan, and highlights implications for (re)training tuberculosis workers in developing countries.

## Introduction

Tuberculosis currently claims about 1.8 million lives yearly, directly causes a US$ 13 billion annual decline in workers productivity, and is one of only several diseases for which specific control targets have been set in the Millennium Development Goals. In tuberculosis control, health workers' calibre and adequacy largely determine program quality and efficiency, as workers consume the bulk of running costs and manage the other resources. The World Health Organization (WHO) *Global Plan to Stop TB 2006 – 2015 *[1] acknowledges that the main human resource issues affecting tuberculosis control are insufficient quality, quantity and distribution of health workers. According to the Stop TB Partnership, $US 250 million is required annually to provide training and technical support to tuberculosis endemic regions. Training of health workers is an important strategy for improving health workers' productivity. Poor performance may be a result of health staff not being sufficient in numbers, or not providing care according to standards, and/or not being responsive to the needs of the community and patients. Apart from training, other influences on productivity of health workers in tuberculosis control include personal and lifestyle-related factors, living circumstances, adequacy of preparation for work during pre-service education; health-system related factors such as human resources policy and planning; job satisfaction related factors such as financial remuneration, working conditions, management capacity and styles, professional advancement and safety at work. These factors constitute a 'productivity mix', of which tuberculosis training is an important component [2].

## Discussion

Many factors encumber the evaluation of the contribution of training to tuberculosis health workers' performance. For example, sub-optimal human resources information systems hamper efforts to determine the adequacy of training and efficiency of workforce management in many tuberculosis control programs. A 2005 World Health Organization (WHO) study to assess workforce capabilities in countries with a high burden of tuberculosis determined that poorly developed human resources information systems compromised the reliability of data on tuberculosis workforce, and that wide variation in training course duration and staff numbers were poorly correlated with tuberculosis programs' performance [3].

Tuberculosis training is incorporated into the basic training curriculum of physicians, nurses, community health officers and laboratory technicians in most developing countries, but the quality of such training varies widely within and between countries. The quality and sustainability of integrated tuberculosis programs depend critically on the extent to which such basic training is of uniformly high quality [4]. Post-basic training for tuberculosis control is inadequately funded by most developing country governments. This is partly due to the small budget for tuberculosis control in most poor countries, the bulk of which is used to pay staff salaries. For instance, over the past three years, government annual budgets for tuberculosis control in Nigeria and Kyrgyzstan averaged US$ 14 million and US$ 1 million respectively, barely enough for staff salaries, let alone training. Another reason for poor funding is that Nigeria and most other tuberculosis endemic countries have so far yet to develop clear staffing needs, which would guide funding agencies to allocate appropriate training budgets. Short NGO-funded tuberculosis training courses are valuable and generously provided to front-line workers. However, the extent to which such well-funded courses positively impact on health worker performance in developing countries is difficult to determine. Most NGO-funded courses offer financial incentives and travelling opportunities for participants that attend, and such incentives have been observed to divert significant human resources away from front-line tuberculosis control duties for considerable periods. They may also lead to inappropriate selection of training participants [5]. The strong influence of NGOs on post-basic specialized tuberculosis programs is beneficial with regard to streamlining the quality of tuberculosis training to a high standard, as well as introducing efficient and innovative learning techniques for tuberculosis training. A recent example of such international training initiatives is the distance learning approach for tuberculosis control doctors and nurses jointly developed in 2006 by the International Council of Nurses, the International Hospital Federation, and the World Medical Association. The globalization of distance education provides many opportunities for developing countries to rapidly scale up tuberculosis training at a fraction of the cost of classroom-based learning approaches. While Internet based distance and open learning approaches are becoming popular, most developing countries still rely heavily on mail correspondence and radio media for distance learning activities.

However, overwhelming influence on tuberculosis training by international donor organizations has a potential to create tensions if NGO priorities are in conflict with the host government training priorities. For example, the International Leprosy Associations' preference for combined Tuberculosis and Leprosy training of frontline staff which resulted in the training of leprosy and tuberculosis control supervisors in Nigeria has a potential to conflict with US Agency for International Development funding for combined tuberculosis and HIV training, which is more in line with the training preferences of the Global Fund to Fight AIDS, Tuberculosis and Malaria.

Of the estimated 8.8 new TB infections in 2005, 7.4 million (84%) were concentrated in Asia and sub-Saharan Africa. Nigeria has the highest TB burden in Africa, fifth highest estimated TB burden worldwide, and is the most populous country in Africa. Kyrgyzstan has the second highest TB burden in Central Asia, after Kazakhstan. Both are developing countries, and are included in WHO's list of 25 priority MDR-TB and XDR-TB countries. Selected demographic and TB-related statistics for both developing countries are shown in Table 1[6].

**Table 1:** TB profiles for Nigeria and Kyrgyzstan, 2005–2006 [6]

	**Nigeria**	**Kyrgyzstan**
**Population**	141.4 million	5.2 million
**Gross national income per capita (US$)**	560	440
**Total health expenditure per capita (US$)**	22	20
**Estimated incidence (all cases/100 000 population/year)**	371 642 (283/100 000 population)	6346 (121/100 000 population)
**Estimated prevalence (all cases/100,000 population/year)**	704 388 (536/100 000)	7013 (133/100 000)
**Estimated mortality (deaths/100,000 population/year)**	99 938 (75/100 000 population)	927 (18/100 000 population)
**Estimation proportion of TB patients with HIV co-infection**	27%	10%
**DOTS case detection rate**	22%	91%
**Total DOTS notification rate**	44/100 000/year	117/100 000/year
**DOTS coverage**	65%	100%
**DOTS treatment success**	59%	82%
**Total registered nurses**	210 923	12 902
**Total registered physicians**	34 923	12 902

The training system for tuberculosis control workers in Nigeria exemplifies tuberculosis training programs in sub-Saharan Africa, while training programs for Kyrgyzstan's tuberculosis workers exemplify training programs in Eastern Europe and Central Asia. Although the structure of the tuberculosis control programs in Nigeria and Kyrgyzstan are similar in the sense in which they currently comprise vertical and integrated components, there are significant differences in the staff structure. First, the Nigerian tuberculosis program may be described as integrated but poorly functioning until 1991 when the National Tuberculosis and Leprosy Control Program was launched and Nigeria's National Tuberculosis and Leprosy Training Centre (NTBLTC) was subsequently established. The NTBLTC focuses on the post-basic training of community health workers as tuberculosis and leprosy control supervisors, the largest cadre of dedicated tuberculosis workers in Nigeria. The centre is also actively involved in the training of laboratory staff on TB diagnosis. Other core cadres of Nigeria's tuberculosis workers – nurses and doctors – have their basic tuberculosis training integrated into their training curriculum, although nursing and medical students in the proximity of NTBLTC undergo TB and leprosy training at the centre. The Postgraduate Medical College of Nigeria conducts training for respiratory physicians, but not specifically for TB specialists, as no such cadre exists in Nigeria. Human resources capacity for effective tuberculosis control in Nigeria remains weak at all levels. Nigeria is currently reforming its tuberculosis training strategy in line with recommendations by the Global Fund and moving towards collaborative HIV/TB training activities. In contrast, the Kyrgyzstan tuberculosis training program may be described as vertical and well functioning until the collapse of the Former Soviet Union (FSU) in 1991. Specialist programs in tuberculosis are integral to the post-graduate curriculum of doctors and nurses in Kyrgyzstan. Doctors are eligible for specialist tuberculosis physician status after a 12-month postgraduate training. Most tuberculosis clinics are staffed by specialist tuberculosis doctors, nurses and laboratory technicians. Kyrgyzstan has maintained strong human resource capacity for tuberculosis control, and the National Tuberculosis Institute (NTI), which is semi-autonomous from the Kyrgyzstan health ministry, is currently reforming its tuberculosis training structure in favour of an integrated model with an emphasis on generalist physicians and nurses accessing quality training on tuberculosis management and playing more active roles in the initiation and continuation of treatment. Tuberculosis physicians constitute the biggest cadre of specialized tuberculosis health workers in Kyrgyzstan. The cadre of Tuberculosis Control Supervisor does not exist in Kyrgyzstan. The progress and problems experienced by the two countries in the training of front-line tuberculosis workers provide ample lessons for improving health workers' training programs in tuberculosis endemic countries.

There are significant training and policy implications if tuberculosis management in a given setting is to operate as a vertical service, a combined service, or as an integrated service. Ideally, training for tuberculosis management should be integrated into the general education and health systems. However, in many tuberculosis-endemic countries, the general education and health systems are too weak to support effective tuberculosis control and training services. Particularly in FSU countries such as Kyrgyzstan, tuberculosis training is highly specialized, and, until recently, was semi-autonomous from the training of other health cadres. NGOs perceive a need to provide interim assistance to rapidly up-skill front line workers through vertical programmes in order to assist with the management of patients already in need of tuberculosis treatment. Such national tuberculosis training and management contexts have encouraged the development and funding of vertical tuberculosis training programmes in many countries until early this decade, despite its limitations [7,8]. In Nigeria, post-basic training of tuberculosis control supervisors is combined with leprosy training. Most of the recurrent funding for such training is obtained from donor members of the International Leprosy Associations. However, as leprosy prevalence in Nigeria continues to decline and as funding shortfalls decimate the public health system, 'reverse integration' of some general health services into better funded leprosy control programs has been occurring in many projects [9,10]. Reverse integration describes the process of bringing other general health services into vertical leprosy programmes. The sharp rise in tuberculosis consultations and treatment in hitherto vertical leprosy projects and general hospitals following the funding of free TB treatment by NGOs has also led to improved funding for tuberculosis training of primary health care providers, thus positioning Nigeria's tuberculosis and leprosy control programs closer in structure to integrated programs. For example, at the NTBLTC in 2006, only 284 outpatient consultations were undertaken for patients with leprosy, compared with 1463 tuberculosis consultations, 8929 general health consultations and 11 564 dermatology consultations [11]. In Kyrgyzstan, the NTI remains focused on the treatment of tuberculosis patients. Post-basic tuberculosis training is conducted within the framework of a vertical tuberculosis control program, but plans are under way to combine tuberculosis with tobacco control in line with the Practical Approach to Lung Health framework, as well as with HIV/AIDS control, in accordance with the WHO interim policy on collaborative HIV/AIDS activities [12,13]. So far, there is no intention by the Kyrgyz NTI to develop a cadre of Tuberculosis Control Supervisors.

There is as yet no international consensus regarding the relative emphasis that should placed on the training of physicians, nurses, laboratory technicians and community health workers in order to produce an optimal human resources mix for tuberculosis control [14], and nations in Central Asia and Eastern Europe, with a strong medical hierarchy in tuberculosis control, are somewhat sceptical about initiatives to 'dilute' the concentration of tuberculosis physicians with nurses and community health workers. Furthermore, few national programs monitor links between tuberculosis (re)training and health worker performance. In this article, differences in the quality, quantity, and distribution of front-line tuberculosis staff in Nigeria and Kyrgyzstan tuberculosis control programs are used to highlight the above training-related issues, and to propose benchmarks for tuberculosis (re)training of frontline healthcare workers in developing countries.

### Quantity

The cost of hiring tuberculosis healthcare workers contributes at least 75% to the total cost of curing a TB patient. It is therefore important that the quantity of health care workers is optimal, as redundant staff will only serve to hike program costs, an unaffordable luxury in developing countries. In this regard, the mix of healthcare workers involved with TB control should maximize human resource capabilities by striking a balance between quality, affordability and program objectives. For instance, a study in Bangladesh showed that using barely literate but motivated and supervised community health workers for DOTS in rural areas halved the total operating cost, and produced similar cure and success rates, compared with an approach using more qualified staff [15]. For a fixed amount of health care dollars, less wastage on human resources costs should translate to more funds for drugs, diagnostic facilities, surveillance and improved patient care. Thus estimates of quantity of frontline tuberculosis workers should focus not just on total numbers, but also on the most cost-effective mix of different cadre of staff.

The 2004 Joint Learning Initiative Report on human resources for health used three categories to identify the density of health workers as low, medium or high: less than 2.5, 2.5–5.0 and 5.0 or more health workers respectively per 1000 population. The average tuberculosis prevalence in developing countries is 3 patients per 1000 population [16]. The authors recommend a mix of 0.125 physician, 0.25 nurse, 0.031 laboratory technician and 0.5 tuberculosis control supervisor working as frontline tuberculosis staff per 1000 population as an optimum human resources mix. This model suggests that one doctor per 24 tuberculosis patients, one nurse per twelve patients, one laboratory technician per 100 patients, and one tuberculosis supervisor per six patients constitute a sufficient human resources mix for front-line tuberculosis staff in developing countries. A 2005 report by the World Bank highlights the difficulties in determining what should be the norm for the number of specialized TB staff in tuberculosis control programs. The ratio for Kyrgyzstan in the same report was one doctor per 17 patients. Kyrgyzstan's TB doctor-patients' ratio is about average for Central Asia, a region with an over-supply of tuberculosis physicians. The same report describes neighbouring Uzbekistan, with one doctor per 14 tuberculosis patients as having "too many doctors" [17].

Recent estimates indicate that Africa has, on average, 2.3 health workers per 1000 inhabitants, and that 36 of 57 countries experiencing substantial shortage of health workers are in Africa. At least 1 million health workers are urgently needed in Africa [18]. Nigeria is currently critically deficient in meeting its workforce requirements in relation to physicians (currently 0.3 per 1000 population), nurses (currently 1.7 per 1000 population), community health workers (currently 0.9 per 1000 population) and laboratory health workers (currently 0.005 per 1000 population). The proportion of Nigeria's health staff actively involved with tuberculosis control is significantly less than the above ratios. For instance, WHO estimates that the TB-specialized physician per population ratio in Nigeria is currently between 1: 160 000 and 1: 400 000 population [19]. In contrast, Kyrgyzstan has 3.0 physicians per 1000 population, 6.1 nurses per 1000 population, 3.7 laboratory workers per 1000 population but practically no tuberculosis community health workers or tuberculosis control supervisors. A comparable proportion of Kyrgyzstan's physicians, nurses and laboratory specialists are employed in tuberculosis control [18,20].

Nigeria's National Tuberculosis and Leprosy Training Centre is responsible for the training of community health officers and nursing staff as tuberculosis control supervisors and district coordinators [21]. The main aim of this training program is to enable participants to effectively implement and monitor tuberculosis treatment guidelines in accordance with the DOTS and DOTS-plus approaches. Despite apparent political commitment shown by the 2001 Abuja Declaration to 'Stop Tuberculosis', Nigeria continues to lag behind in its target of training at least one nurse or community health worker as a Local Government Area tuberculosis and leprosy control supervisor/coordinator in each of Nigeria's 774 local government areas. Unfortunately, the biggest funding shortfall in Nigeria's tuberculosis budget has consistently been in the area of training to improve case detection and cure rates [19,22].

In Kyrgyzstan, there is a surfeit of front-line tuberculosis staff with the exception of District Tuberculosis Control Supervisors/Coordinators. Kyrgyzstan workforce is skewed towards high-cost specialist tuberculosis physicians, in line with the hospital-based organization of tuberculosis services in the FSU. The low salaries of Kyrgyzstan's tuberculosis and other health workers (average US$ 100 per physician per month) remain a major demotivating factor for improving productivity. In post-Soviet Kyrgyzstan, fiscal constraints, limited lucrative employment opportunities for new tuberculosis physicians, and health sector reforms have al had major effects in reducing the number of physicians, nurses and laboratory workers trained at tertiary institutions, as well as the number of tuberculosis physicians employed by the public sector tuberculosis treatment facilities.

### Quality

Quality of training has a strong influence on the quality of care provided to patients. Quality of care may be defined as the degree to which health services for individuals and populations increase the likelihood of desired health outcomes and are consistent with current professional knowledge. Process indicators of quality of tuberculosis control entail the assessment of what the health care provider did for the patient and how it was done. These indicators measure the activities and tasks in patient episodes of care [23]. Developing quality benchmarks for tuberculosis training and health workers performance should be accorded high priority given wide variations in tuberculosis training curricula in high burden countries [3]. Current approaches of quality evaluation of health care workers for TB control appear to focus on the extent to which countries meet the WHO targets of diagnosing at least 70% of new smear positive cases, and curing at least 85% of such cases. The use of these indicators alone will position Kyrgyzstan as operating a high-quality TB programme with commendable health worker performance, while the quality and health care workers' performance of Nigeria's TB programme will be deemed as unsatisfactory. However, the sole use of WHO indicators may mask other factors that contribute to meeting case detection and cure objectives.

Although concerted efforts have been made to improve the quality of tuberculosis training and quality control for laboratory staff [24], the main beneficiaries of these quality improvement practices have been staff and programs of developed countries. In Nigeria, data quality for AFB microscopy for relatively well-funded leprosy programs is poor [25], and the quality of poorly funded tuberculosis laboratory services in Nigeria is probably worse. Kyrgyzstan has a better quality laboratory service, and its laboratory services are better quality-controlled compared with most developing countries. In order to streamline the quality of tuberculosis training in developing countries, it is suggested that minimum standards for national training curricula for frontline staff should be developed (and revised regularly) in consultation with WHO, International Union Against Tuberculosis And Lung Disease (IUATLD) and the respective regulatory bodies for the training of physicians, doctors and laboratory technicians. Such training was conducted in a haphazard fashion in Nigeria's nursing schools until early in the 21^st ^century, leading to generally poor knowledge and negative attitude of nurses vis-à-vis leprosy and tuberculosis patients [26]. In recent years, institutional reforms have been implemented, and tuberculosis and leprosy training is currently a core part of Nigeria's nursing curriculum [27]. Also pertinent is the need to develop standard, multi-disciplinary training programs for quality assurance of tuberculosis control programs, such as that developed by Netherlands' Royal Tropical Institute [28].

Apart from quality assurance of training programs at the curriculum development level, it is also necessary to monitor quality of tuberculosis training at participant learning, job behaviour and organizational levels. Participant feedback, pre-test, post-test and performance tests (e.g. role play and evaluation of reports during training) are useful quality tools at participant learning level. Tools for assessing quality of training at job behaviour level include questionnaire studies of participant's impressions of how the training is impacting on job performance, and formal site visits by trainers to observe participants at work settings. At the organizational level, quality of training may be indirectly assessed by its impact on case detection, treatment outcomes, and validity of reports from tuberculosis control programs.

In Nigeria, refresher training for specialized and primary care staff responsible for tuberculosis control has not been adequately addressed, apart from the cadre of tuberculosis control supervisors. Although undergraduate medical, nursing and laboratory technicians' training programs include tuberculosis topics, the quality of such training tends to vary with the enthusiasm of tuberculosis specialists at individual training institutions. The proportion of medical and nursing staff engaged in government-funded post-basic tuberculosis training at teaching hospitals is currently too small relative to Nigeria's tuberculosis control needs.

Kyrgyzstan's National Tuberculosis Institute coordinates post-graduate physician training, which typically lasts twelve months. Tuberculosis physicians are required to undertake government-funded mandatory update courses at the National Tuberculosis Institute once every three years. Most of the update courses on tuberculosis for non-specialist tuberculosis physicians in Kyrgyzstan are currently funded by NGOs. Nursing and laboratory workers also have the opportunity to undertake high quality training programs funded by International NGOs involved with tuberculosis control, such as Medecins Sans Frontiers, United States Agency for International Development, and Project Hope. The recent health system reforms which resulted in a reduction in the number of government laboratories and laboratory staff, increased provision of modern equipments and adequate reagents to regional laboratories and more frequent training of tuberculosis physicians, nurses and laboratory staff have strong potential for improving the quality of tuberculosis services in Kyrgyzstan.

### Distribution

Tuberculosis being a disease that is strongly influenced by poverty, living conditions, and co-morbidities such as HIV infection, its distribution within nations is usually uneven. Since failure of control measures is an important determinant of the distribution and spread of tuberculosis [29], it is important to focus trained human resources in areas of high tuberculosis prevalence. Unfortunately, accurate data on the distribution of tuberculosis workers in developing countries is sparse, and this data deficiency requires urgent attention. Nevertheless, anecdotal evidence indicate that the geographical areas in which tuberculosis prevalence outstrips distribution of trained and experienced tuberculosis health workers are poor rural and urban areas, and prisons.

Data for TB prevalence in poor rural areas of most developing countries are inaccurate, and most underestimate the TB burden remote regions due to low case detection. The urban slums of Moscow are populated by poor migrant workers from Kyrgyzstan and other Central Asian countries, many of whom contract tuberculosis due to congested living conditions and limited access of infected migrant workers to treatment [30]. Currently, about 60% of the population of Nigeria and Kyrgyzstan reside in rural areas. In prison settings in both countries, TB prevalence is demonstrably higher compared with the general community. For instance, the TB prevalence in Kyrgyzstan's prisons was estimated by World Bank epidemiologists at 5500 per 100 000 prisoners. This is more than 47 times as high as the TB prevalence in the general community [17]. Ideally, health ministries and tuberculosis program managers should endeavour to correlate the distribution of tuberculosis health workers with the prevalence of tuberculosis in the community. Planning for a good match between human resources needs and disease prevalence in prisons and poor rural and urban settings need to begin from incentives provided during the basic training of physicians, nurses, and laboratory technicians. While preferential allocation of candidates raised in rural areas to training slots may sometimes act as an incentive for such candidates to return to rural areas following completion of their training, the majority of qualified health workers that currently work in rural areas were raised in urban areas. Thus, the enhancement of quality of rural experiences during undergraduate postings, and the promotion of the challenges and lifestyle of rural practice to health workers may play important roles in encouraging health workers to work in underserved rural areas [31,32].

In Kyrgyzstan, a remarkable government incentive to encourage tuberculosis health workers to work in prisons is a legislation giving such workers access to pension benefits in half the time it takes for workers posted to other areas. They also enjoy disability insurance in the event of contracting tuberculosis. In some rural regions, a TB specialist is paid US$ 2.20 for each patient diagnosed and cured. Such targeted performance-based funding of health workers has contributed to equitable distribution of health workers in Kyrgyzstan [17,33].

In Nigeria, limited non-monetary incentives, such as short-term overseas training are provided to Nigerian front-line medical staff who have worked in tuberculosis control for several years, and who accept to work in tuberculosis control for at least several more years following their training. Salaries and working conditions in Nigeria for health workers are poor and the stigma as well as poor infection control measures which magnify a risk of infection with multi-drug resistant tuberculosis make the tuberculosis control sector less likely to attract and retain qualified and dedicated staff. For instance, in 2006, only 62 new TB supervisors were trained at the NTBLTC, and a shortfall of at least 200 tuberculosis supervisors urgently needs to be bridged. The majority of these supervisors are needed in rural and underserved areas such as prisons. Unlike the situation in Kyrgyzstan, the Nigerian government is no longer the most significant employer of health workers in rural and high prevalence tuberculosis regions of Nigeria, as NGOs and the private health sector continue to expand due to poor conditions of service in the public sector, and limited incentives for health workers to work in tuberculosis control in rural areas and prisons. Notification of tuberculosis treatment by private practitioners and NGOs in Nigeria is unsatisfactory, thus hampering efforts to determine the impact of these workers in controlling tuberculosis in remote and other tuberculosis high prevalence areas. Annual tuberculosis training budgets provided by Nigerian governments are consistently grossly inadequate, making it especially difficult to utilize training incentives to recruit and retain front-line workers in understaffed regions.

It must however be emphasized that just getting tuberculosis workers to the 'right' work location may not be enough to improve tuberculosis control outcomes. Especially in rural and prison settings, it is also important to facilitate access to health facilities by patients, provide staff with adequate transport, and encourage outreach activities whereby tuberculosis health workers in rural areas can actively seek their patients instead of passively waiting in health centres in order to optimize the use of available services and skills. In prison settings, apart from retaining a high calibre and sufficient quantity of tuberculosis workers, it is equally important for such health workers to have adequate access to tuberculosis patients, and to have the drugs and equipments to facilitate optimal treatment.

The distribution of health workers in given settings is strongly influenced by staff preferences, but tuberculosis training programs may be used as an incentive to influence health workers' distribution patterns. Such influences include preferentially allocating funded local and international tuberculosis training places to eligible applicants from geographical regions with relatively high disease burden, and making fully-funded tuberculosis training conditional on trainees working in a tuberculosis high prevalence region for a specified period. Prisons constitute an epidemiological pump for tuberculosis transmission in most endemic countries, and thus require a fair share of high quality tuberculosis staff. Given the difficulties that specialist staff are likely to face with regards to living conditions in some high tuberculosis prevalence settings such as prisons and rural areas, it may be prudent to factor prison/rural posting package costs (e.g. relocation allowance, subsidized housing, 'environmental allowance) into conditional training programs in order to enhance staff retention and motivation [5,34].

Because Kyrgyzstan has adequate (in fact surplus) numbers of tuberculosis physicians, and because the vast majority are employed within the public sector, ensuring adequate distribution through government employment policies and directives has so far been a relatively easy obstacle to surmount. NGOs have taken active roles in improving the 'productivity mix' for the country's prison system and in community settings, in line with international benchmarks [35,36]. There is a need to plan for the training of low-cost staff, who would be more likely to provide tuberculosis treatment services at a much lower cost in this mountainous nation.

In contrast, since Nigeria's cadre of skilled tuberculosis health workers is grossly inadequate for the country's requirements, systematic interventions to facilitate the optimal distribution of scarce human resources are urgently required. National and State tuberculosis programs currently lack sufficient incentives and authority to influence the distribution of physicians, nurses and laboratory technicians skilled in tuberculosis control. The only cadre over which there is some measure of control are the community health workers, most of who are sponsored by NGOs and local government councils and who are expected to return to their respective local government areas after completing their training. However, the distribution and absolute numbers of this cadre is still inadequate to meet the country's needs. Lack of clear career prospects for TB supervisors is making retention of this cadre of staff difficult. The quantity, quality and distribution of skilled tuberculosis workers in Nigeria's prison settings are grossly inadequate. Nigeria's prisons remain a major source of tuberculosis transmission among prisoners, and from prisoners to the larger community.

## Conclusion

Appropriate (re)training of front-line health workers is a necessary but not sufficient activity for improving health worker performance as well as the quality of tuberculosis control outcomes. This review underscores the need for tuberculosis policy makers, professional bodies, and NGOs working in developing countries to address the following training-related issues:

• Trained human resources operate in a 'productivity mix' comprising other factors such as adequate motivation and incentives, availability of required chemotherapy and supplies for appropriate patient care, and retention of qualified health staff in high tuberculosis-prevalent areas. As more funds become available for training, it is important to pay attention to other factors of the 'productivity mix', so as not to reinforce the limitations and weaknesses of current training practices in developing countries. Other African and Central Asian countries that have addressed most facets of the TB 'productivity mix', such as Malawi and Kazakhstan, continue to record significant improvements in TB case detection and cure rates [17,37,38].

• Planning for training needs of tuberculosis control programs requires a good human resources information system, which is currently poorly developed in countries with a high burden of tuberculosis [3]. It is important to adopt an internationally coordinated approach to addressing this deficiency, and international non-governmental organizations are best positioned to fund human resources information systems with comparable datasets across developing countries. Particular attention should be paid to adequate data collection on the distribution of health workers generally, and front-line tuberculosis workers in particular. In Nigeria and other developing countries with strong private sector involvement in tuberculosis management, greater surveillance coordination is required with regards to case detection and treatment outcomes.

• More can and should be done by governments and training regulatory authorities in developing countries to improve on the quality of  tuberculosis education during the basic training of nurses, doctors and  laboratory technicians. While most developing countries inadequately fund post-basic training of tuberculosis workers, more can be done by governments and training regulatory authorities in these countries to improve on the quality of tuberculosis training during the basic training of nurses, doctors and laboratory technicians. Such improvements may entail coordinated national approaches to incorporate tuberculosis training as core aspects of the curricula of front-line tuberculosis workers, and the provision of training incentives to teaching staff in medical, nursing and laboratory technology schools to improve the quality of training at these levels. A system for evaluating the quality of tuberculosis training at this level also needs to be developed.

• Most post-basic tuberculosis training in developing countries is funded by international NGOs. As such, these agencies have a strong influence on the structure of TB training (e.g. combination of TB training with leprosy, respiratory diseases and/or HIV/AIDS), as well as the mix of health worker cadres that will be trained in order to efficiently undertaken tuberculosis control services. Incentives for training at this level have a strong influence on the distribution of tuberculosis health workers in developing countries. It is important to minimize the adverse impact of training on the availability of health workers in high-need areas by developing, funding and promoting distance learning and on-site training programs. Distance learning is not necessarily synonymous with Internet-based learning, since postal correspondence courses and narrow-cast radio and television media may be used to supplement on-site clinical training for improving knowledge and skills for tuberculosis control [39,40].

• There is a need to standardize the training curriculum for post-basic training of staff in developing countries. As noted by a recent WHO survey [3], wide variations in training duration and structure are poorly correlated with program performance. WHO, tuberculosis associations, research and training institutes, and international NGOs need to play a more pro-active role in working with national tuberculosis programs to develop and implement standardized training curricula for front-line tuberculosis workers.

• It is important to evaluate the contribution of training to improving health workers' productivity and the quality of tuberculosis control programs. Such evaluation should occur at three levels: (a) During training, through feedback from participants, quality of written and practical training-related assignments undertaken by participants, and pre-test/post-test evaluations; (b) within twelve months following training, through the use of questionnaires to facilitate participants' assessment of the impact of training on their performance, as well as site visits by trainers, to observe participants in clinical and field conditions; (c) evaluation of tuberculosis program outcomes, with particular attention to improvements in case detection rates and cure rates.

## Declaration of competing interests

The authors declare that they have no competing interests.

## Authors' contributions

NA conceived of the study and participated in its design and coordination. IS provided information on Kyrgyzstan tuberculosis training program and working conditions of Kyrgyz tuberculosis staff. AD provided information on Nigeria's tuberculosis training program and working conditions of Nigeria's tuberculosis field workers. All authors read and approved the final manuscript.
